# Seasonal changes in the altitudinal distribution of nocturnally migrating birds during autumn migration

**DOI:** 10.1098/rsos.150347

**Published:** 2015-12-09

**Authors:** Frank A. La Sorte, Wesley M. Hochachka, Andrew Farnsworth, Daniel Sheldon, Benjamin M. Van Doren, Daniel Fink, Steve Kelling

**Affiliations:** 1 Cornell Laboratory of Ornithology, Cornell University, Ithaca, NY 14850, USA; 2 College of Information and Computer Sciences, University of Massachusetts, Amherst, MA 01003, USA; 3 Department of Computer Science, Mount Holyoke College, South Hadley, MA 01075, USA

**Keywords:** flight altitude, mid-latitudes, polar-front jet stream, seasonal avian migration, weather surveillance radar, wind

## Abstract

Wind plays a significant role in the flight altitudes selected by nocturnally migrating birds. At mid-latitudes in the Northern Hemisphere, atmospheric conditions are dictated by the polar-front jet stream, whose amplitude increases in the autumn. One consequence for migratory birds is that the region’s prevailing westerly winds become progressively stronger at higher migration altitudes. We expect this seasonality in wind speed to result in migrants occupying progressively lower flight altitudes, which we test using density estimates of nocturnal migrants at 100 m altitudinal intervals from 12 weather surveillance radar stations located in the northeastern USA. Contrary to our expectations, median migration altitudes deviated little across the season, and the variance was lower during the middle of the season and higher during the beginning and especially the end of the season. Early-season migrants included small- to intermediate-sized long-distance migrants in the orders Charadriiformes and Passeriformes, and late-season migrants included large-bodied and intermediate-distance migrants in the order Anseriformes. Therefore, seasonality in the composition of migratory species, and related variation in migration strategies and behaviours, resulted in a convex–concave bounded distribution of migration altitudes. Our results provide a basis for assessing the implications for migratory bird populations of changes in mid-latitude atmospheric conditions probably occurring under global climate change.

## 1. Introduction

Seasonal avian migration is a diverse phenomenon whose patterns and drivers have been examined across multiple spatial and temporal scales and biological perspectives [1]. One area in which our understanding is limited is the way in which migrants associate with atmospheric conditions during migratory flight. This knowledge gap originates from the challenge of acquiring accurate information on the vertical location of migrants, and is most acute for small-bodied species, the majority of which migrate at night and at high altitudes. Recent technological advancements in radar-image processing have overcome some of these limitations [2]. Analyses of radar derived data indicate that nocturnal migrants occur in high concentrations up to *ca* 3000 metres above sea level (m.a.s.l.), with migration altitudes often centred at *ca* 900 m.a.s.l. [3–9]. Current evidence suggests migratory birds tend to associate with altitudes that contain mild winds that are primarily supportive [3,5,10], but in some cases opposing [11,12]. Additional evidence suggests migrants ascend to the lowest altitudes at which atmospheric conditions first become supportive, even when more optimal conditions may exist at higher altitudes [7,9]. A common feature shared across these studies is that altitudinal distributions are rarely examined longitudinally, where information is compiled and analysed across the migration season (but see [8]). The lack of a longitudinal perspective has the potential to result in partial or incomplete inferences, especially in geographical regions where seasonal fluctuations or trends in atmospheric conditions may influence how migratory behaviour is defined across the season.

**Figure 1.**
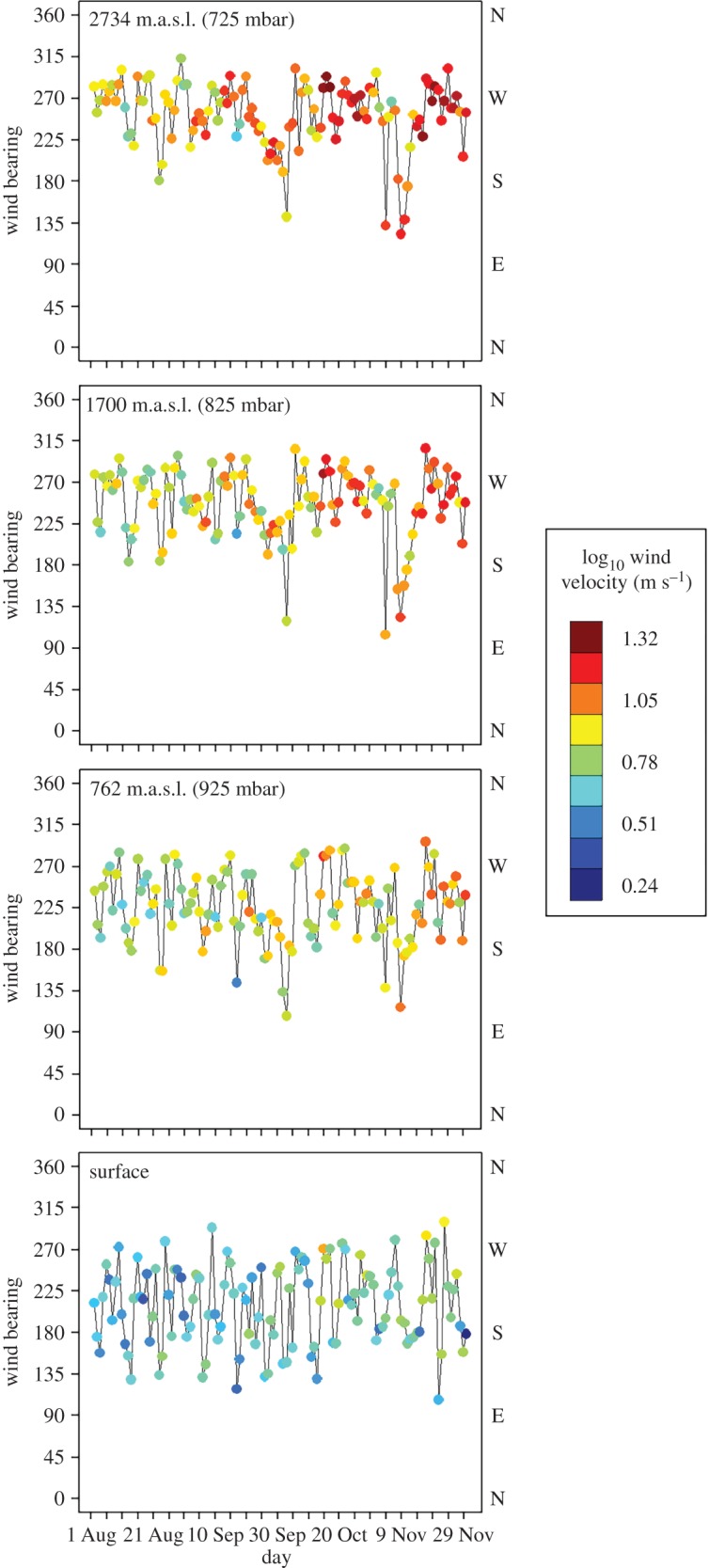
The bearing of origin and velocity of nocturnal winds estimated daily at 30 m above ground level (surface) and at three isobaric levels (925, 825 and 725 mbar) representing altitudes of *ca* 762, 1700 and 2734 m.a.s.l., respectively. Values are averaged within a 37.5 km radius circle of 12 weather surveillance radar stations located in the northeastern USA (see the electronic supplementary material, figure S1) during the autumn of 2010 and 2011 combined.

The mid-latitudes of the Northern Hemisphere during autumn migration is one such region, containing variable and systematic trends in atmospheric conditions, as well as being the region hosting the majority of the world’s migratory bird species during migration and the breeding season [13]. A major source of variation at mid-latitudes in atmospheric conditions is the polar-front jet stream, a narrow high-speed air current located between 7 and 12 km a.s.l. (*ca* 250 mbar) that flows west to east whose path defines broad meanders that extend north to south [14,15]. The polar-front jet stream is driven primarily by the difference in temperature between the Arctic and mid-latitudes. As the magnitude of these differences changes seasonally, the location, velocity and amplitude of the jet stream responds accordingly. The smaller temperature difference between the Arctic and mid-latitudes during the summer results in a slower jet stream with lower amplitude whose meridional flow is constrained to 50–60^°^ N latitude. During the winter, the temperature differences between the Arctic and mid-latitudes increases, resulting in a faster jet stream with higher amplitude whose meridional flow can extend as far south as 30^°^ N latitude. A primary consequence for nocturnally migrating birds travelling through the mid-latitudes in the autumn is that the prevailing westerly winds within the region become progressively stronger at higher migration altitudes, resulting in conditions that may interfere with southward migration (figure 1).

There is evidence that nocturnal migration intensity within mid-latitudes in North America is greatest when wind speeds at migration altitudes are mild [16]. Therefore, as the autumn migration season progresses and winds become increasingly stronger at higher migration altitudes (figure 1), an initial expectation is that migration altitudes should decline on average. More specifically, in order to avoid strong crosswinds that may interfere with southward migration journeys [7,9], we expect migrants to occur at increasingly lower flight altitudes as the season progresses. Additionally, there is evidence that species composition of nocturnal migrants within the region transitions in the autumn from small- to large-bodied migratory species [16]. Owing to the generally poor climbing performance of large-bodied species, especially during nocturnal flight [17,18], we expect late-season large-bodied migrants to occur in greater concentrations at lower migration altitudes, thus adding support to our prediction. There is also evidence that species composition of nocturnal migrants within the region transitions in the autumn from long- to short-distance migratory species [16]. In order to associate with lower air densities and more supportive atmospheric conditions [19], early-season long-distance migrants may expend the additional energy to climb to higher migration altitudes [17,18]. Late-season short-distance migrants, by contrast, are more likely to remain at lower flight altitudes to maintain the overall efficiency of their migration journeys [17,18], especially late in the season when strong winds at higher migration altitudes are more probable (figure 1). Thus, flight altitudes of long-distance migrants that migrate early in the season and short-distance migrants that migrate late in the season may provide additional support to our prediction. In sum, differences in the costs of migratory flight based on energy, time and risk as determined by body size and total migration distance [16] may result in associations with decreasing flight altitudes as the migration season progresses, which supports our initial expectation.

To test our predictions, we use reflectivity data from 12 weather surveillance radar (WSR) stations located at mid-latitudes within the northeastern USA (see the electronic supplementary material, figure S1). We estimate the density of nocturnally migrating birds within 100 m altitudinal bins at a daily temporal resolution from 1 August to 30 November for the years 2010 and 2011 combined [20]. To infer how the composition of migratory species changes at the 12 WSR stations over the migration season, we use assemblage-level estimates of body size and total migration distance. We derive these from weekly estimates of diurnal probability of occurrence [21] for 142 species of nocturnal migrants using occurrence information from the eBird database [22]. We expect that documenting seasonal altitudinal distributions at mid-latitudes will provide insights into how nocturnally migrating birds associate with seasonally fluctuating atmospheric conditions, and the implications for migratory bird populations as the form and structure of this seasonal atmospheric behaviour transitions under global climate change.

## 2. Material and methods

### 2.1 Radar data preparation and analysis

We obtained raw Level II NEXRAD Doppler radar data (WSR-88D) for 12 WSR stations located in the northeastern USA (see the electronic supplementary material, figure S1) from the US National Climatic Data Center archive. Detailed methodology for the preparation and analysis of these data is available from Farnsworth *et al.* [20], which we briefly summarize here. During the period from 1 August to 30 November for 2010 and 2011, we retained one radar scan per hour, starting with the scan occurring closest to sunset and then selecting scans as close as possible to 1 h increments after sunset, using civil twilight to define sunrise and sunset. We acquired a total of 39 840 hourly radar scans for our study period. We previewed the hourly radar scans using a custom-designed application and eliminated scans that were contaminated by non-biological targets (e.g. precipitation or ground clutter) or showed ambiguous patterns indicative of atmospheric instabilities. This was implemented within a 37.5 km radius of each WSR station and up to an altitude of 3000 m above the radar station, which encompasses the range of altitudes at which nocturnal migrants have historically been detected by WSR in the region [23]. We retained 22 918 hourly scans for further analysis. We converted the WSR-88D units of reflectivity factor (*Z*, mm^6^ m^−3^) to reflectivity (*η*, cm^2^ km^−3^), a quantity that is easier to interpret biologically [24]: the total effective scattering area of targets per unit volume. To distinguish birds from wind-borne targets such as dust, smoke and insects, we compared radial target velocity with the radial wind velocity at the same location. Reflectivity was summarized within 30 100 m altitudinal bins by averaging each bin’s reflectivity values. We used the elevation of each WSR station (see the electronic supplementary material, figure S1) and the elevation of the 100 m altitudinal bins at each WSR station to convert the altitude of the 100 m bins to altitude a.s.l. This procedure placed the altitudes of the 100-m bins on the same scale across the 12 WSR stations, supporting our analysis of longitudinal trends and atmospheric associations among WSR stations.

We estimated nocturnal atmospheric conditions at a daily temporal resolution for each of the 12 WSR stations during the autumn of 2010 and 2011 using five gridded North American Regional Reanalysis (NARR) three hourly datasets, which resulted in a total of eight variables for analysis (dataset number ds608.0) [25]. The eight variables were wind velocity and bearing at 30 m above ground level, and wind velocity and bearing at three isobaric levels (725, 825 and 925 mbar) representing altitudes of *ca* 2734, 1700 and 762 m.a.s.l. at the 12 WSR stations, respectively (see the electronic supplementary material, figure S2) [26]. The eight variables are spatially organized using the National Centers for Environmental Prediction Grid 221 (Regional North American Grid—Lambert Conformal) grid arrangement (*ca* 32 km spatial resolution). Using methods previously described [27], the eight NARR variables were first summarized for each nocturnal period by averaging values across the three hourly intervals whose times occurred after sunset and before sunrise. The variables were then summarized for each of the 12 WSR stations by averaging across the NARR grid cells whose centres were located within 37.5 km of each WSR station. Wind velocity was log_10_ transformed before averaging. Average wind bearing was calculated using the circular mean.

### 2.2 Analysis of altitudinal distributions

To summarize each evening’s WSR scans for analysis, we calculated the average reflectivity by day, year and WSR station, and average altitude by day, year and WSR station. We used the altitude of the centres of the 100 m altitude bins in these calculations, measured as a.s.l. We used log-transformed reflectivity in these calculations, and we only included reflectivity values greater than 0. This procedure, therefore, summarized the entire altitudinal distribution of reflectivity across 100 m altitudinal bins for each day, year and WSR station. We summarized how these daily estimates of migration altitude changed across the autumn migration season using weighted generalized additive mixed models (GAMMs) [28] fitted using a boosting algorithm based on the component-wise univariate base-learners procedure [29] with year and WSR station as random effects. We fitted the weighted GAMMs to seven quantile levels (*τ*=0.01, 0.05, 0.10, 0.50, 0.90, 0.95 and 0.99) using reflectivity estimated within the 100 m altitude bins as weights to take into consideration altitudinal variation in migration density. Fitting the seven quantile levels allowed us to assess the degree of evidence for heteroscedasticity, i.e. if the variance of daily distribution of migration altitudes changed with time. We estimated uncertainty at each quantile level using point-wise bootstrap 90% confidence intervals (CIs) with 1000 bootstrap samples. We selected 90% CIs to allow for a broader consideration of biological significance. Separate bootstrap samples were generated for each combination of year and WSR station, which were then combined to create a single bootstrap sample for analysis. To determine how seasonal altitudinal distributions of migrants varied across space, we implemented for each WSR station the same weighted GAMM procedure fitted to three quantile levels (*τ*=0.01, 0.50 and 0.99) with year as a random effect.

To determine how the distribution of migration altitudes was associated with atmospheric conditions during autumn migration across WSR stations, we used regression tree analysis combined with linear mixed models [30]. This procedure combines the structure of mixed effects models with the flexibility of tree-based estimation methods. Here, we examined the relationship between reflectivity and wind velocity and bearing separately at three isobaric levels (725, 825 and 925 mbar) using the data sources described above. To support our analysis, we compiled the radar reflectivity data within 200 m altitudinal bands, which approximately encompasses the range of altitudes at the three isobaric levels where wind velocity and bearing were measured (see the electronic supplementary material, figure S2). In the regression tree models, we log-transformed reflectivity to improve its distributional properties, and we included day as a continuous fixed effect, year as a categorical fixed effect and WSR station as a random effect. Thus, the linear mixed model for each isobaric level has the form: reflectivity ∼ velocity + bearing + day + year + station, where velocity, bearing and day are continuous fixed effects, year a categorical fixed effect and station a random effect.

### 2.3 Nocturnal migrant occurrence analysis

We inferred the composition of migratory bird species at the WSR stations using modelled estimates of probability of occurrence for North American birds. Probabilities of occurrence were calculated using spatio-temporal exploratory models (STEM) [21] and occurrence information from the eBird citizen-science database [22] for 448 species during the combined period 2004–2011. The STEM estimates of probability of occurrence were rendered at 130 751 geographically stratified random (SRD) points distributed within a geographical coordinate system at a density of *ca* 15 per 30×30 km cell within the contiguous USA. STEM estimates are defined as the probability that a typical eBird participant will detect a given species on a terrestrial search from 07.00 to 08.00 h while travelling 1 km on the given day at the given location [21]. From these SRD points, we selected those that occurred within a 37.5 km radius of the 12 WSR stations. This resulted in an average of 68 SRD points (s.d.=14) per WSR station. Using previously described methods [27], we applied a threshold procedure to each species to remove very low estimates of probability of occurrence from consideration.

We used body size and total migration distance to summarize how the composition of nocturnally migrating birds changed across time at the 12 WSR stations. These measures have been identified as having important associations with migratory behaviour based on theoretical considerations and empirical evaluations [16,31]. We acquired body size estimates from Dunning [32], which we averaged across sexes and subspecies. Total migration distance was estimated for each species using the procedure described previously [31]. Here, we converted NatureServe Western Hemisphere range maps [33] to an equal-area icosahedron (12 452 km^2^), and calculated the great circle or orthodromic distance between the geographical centroids of the breeding and non-breeding ranges for each species. From the 448 species, we selected for analysis those that were primarily nocturnal migrants, that had probabilities of occurrence greater than 0 during the study period within at least one of the 37.5 km radius circles and had migration distances greater than 0. Following this procedure, 142 species were retained for analysis (see the electronic supplementary material, table S1).

Using these data sources, we generated weekly assemblage-level estimates of body size and total migration distance for each WSR station. Our assemblage-level estimates were calculated using weighted averages where weights were the STEM derived estimates of probability of occurrence of migrants at the SRD points associated with each WSR station. We examined how weighted averages for body size and total migration distance changed across time (weeks) among WSR stations using GAMM with WSR station as a random effect. We fitted GAMMs separately for species in three taxonomic groups classified by the avian orders Anseriformes (*n*=12), Charadriiformes (*n*=17) and Passeriformes (*n*=100; see the electronic supplementary material, table S1). Owing to small sample sizes, the remaining 13 species, belonging to six additional avian orders, were placed into a fourth category, which we labelled ‘other’ (see the electronic supplementary material, table S1). In total, this procedure assumes that diurnal, terrestrial observations of migratory species within the region can be used to accurately estimate trends in assemblage-level body mass and total migration distance across the season.

We conducted all analyses in R v. 3.1.2 [34]. Weighted GAMM was implemented using the gamboost function in the mboost library [35] with a base d.f. =5 for the smoothing spline, 10 000 initial boosting iterations and a shrinkage parameter of 0.01. The regression tree analysis was implemented using the REEMtree library and the REML method to estimate the linear model [30].

## 3. Results

**Figure 2.**
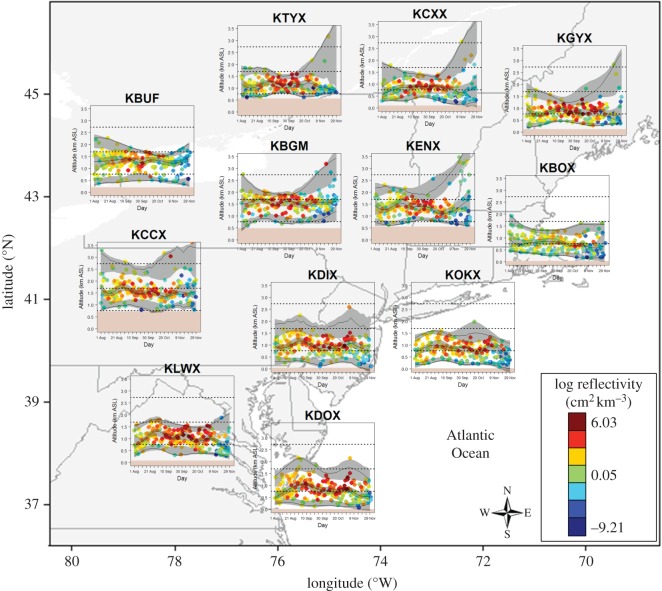
The average altitude (a.s.l.) and reflectivity of nocturnal migratory birds within 100 m altitudinal bins detected at 12 weather surveillance radar stations located in the northeastern USA (see the electronic supplementary material, figure S1) estimated daily during the autumn of 2010 and 2011. The corresponding fit of weighted generalized additive mixed models using reflectivity as weights and year as a random effect to three quantile levels (*τ*=0.01,0.50 and 0.99) with point-wise bootstrap 90% CIs. The dotted lines are the three altitudes considered in the regression tree analysis (762, 1700 and 2734 m.a.s.l.) and the brown band identifies the elevation of each WSR station (see the electronic supplementary material, figure S1).

During the autumn of 2010 and 2011, the seasonal altitudinal distributions of migratory birds showed strong spatial variation among the 12 WSR stations (figure 2). The greatest density of migrants occurred during a similar period in the middle of the migration season, and associations with altitude during this period tended to be more narrowly defined and less variable (figure 2). During the beginning of the season, associations with altitude tended to be more variable for inland WSR stations (figure 2). During the end of the season, inland WSR stations showed more variable associations with altitude, especially WSR stations located in the northern and central portions of the study area (figure 2).

Across the season, the vertical location of the altitudinal distribution of migratory birds was closely associated with the elevation of the terrestrial surface; i.e. migratory birds tended to occur at higher altitudes at higher elevation WSR stations located within the Appalachian Mountains, and at lower altitudes at lower elevation WSR stations located near the Great Lakes or Atlantic Ocean (figure 2; see the electronic supplementary material, figure S3). This positive association between migration altitude and the elevation of the WSR station resulted in altitudinal distributions being more similar among the WSR stations when examined relative to the elevation of the WSR station, especially at lower altitudes (figure 2; see the electronic supplementary material, figure S3).

**Figure 3.**
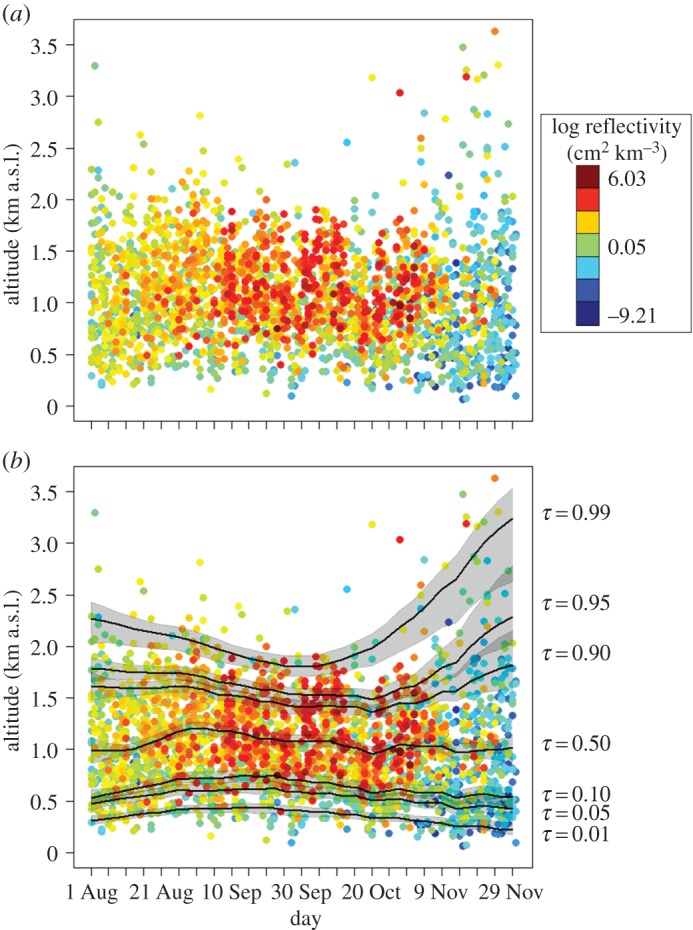
(*a*) The average altitude (a.s.l.) and reflectivity of nocturnal migratory birds within 100 m altitudinal bins detected at 12 weather surveillance radar stations located in the northeastern USA (see the electronic supplementary material, figure S1) estimated daily during the autumn of 2010 and 2011. (*b*) The corresponding fit of weighted generalized additive mixed models using reflectivity as weights and year and WSR station as random effects to seven quantile levels (*τ*=0.01, 0.05, 0.10, 0.50, 0.90, 0.95 and 0.99) with point-wise bootstrap 90% CIs.

When the 12 WSR stations were considered in combination, the greatest densities of nocturnally migrating birds occurred during the middle of the season at altitudes from *ca* 500 to 2000 m.a.s.l. (figure 3*a*). The median migration altitude showed minor fluctuations across the season, remaining near *ca* 1000 m.a.s.l. with no evidence of a seasonal trend (figure 3*b*). However, there was evidence for seasonal variation in both the lower and upper quantiles (figure 3*b*). The lowest three quantiles presented slightly concave altitudinal associations across the season, and the highest three quantiles presented strongly convex altitudinal associations across the season (figure 3*b*). This heteroscedasticity in the distribution of migration altitudes was defined by greater variance early and especially late in the season, forming a convex–concave bounded distribution. Periods of higher variances were associated with greater concentrations of migrants at low and especially high migration altitudes (figure 3*b*). The concentration of high-altitude migrants late in the season was more pronounced, and included the highest migration altitudes documented during the study period (figure 3*b*).

**Figure 4.**
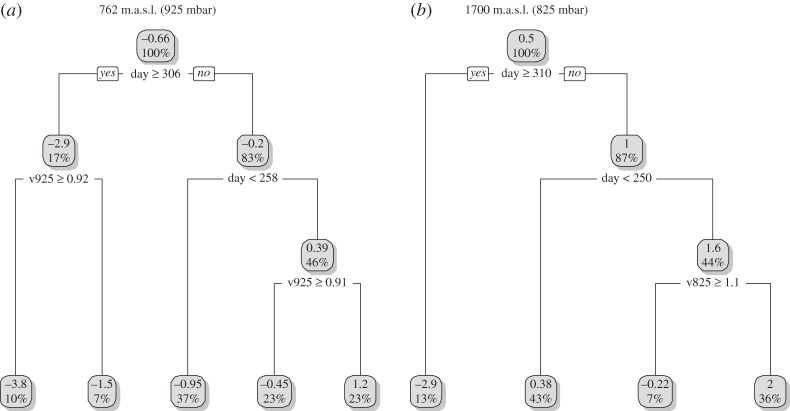
Regression trees predicting daily reflectivity (log-transformed) within 200 m altitudinal bands centred at altitudes of (*a*) *ca* 762 m.a.s.l. and (*b*) *ca* 1700 m.a.s.l. from 12 weather surveillance radar stations (see the electronic supplementary material, figure S1). The predictors include day (1 August to 30 November), year (2010 and 2011), and wind velocity and bearing estimated at 925 and 825 mbar, respectively. The boxes display the average reflectivity and the percentage of observations arriving at each node.

The three 200-m altitudinal bins considered in the regression tree analyses centred at *ca* 762, 1700 and 2734 m.a.s.l. sampled the seasonal distribution of migratory birds differently across the 12 WSR stations (figure 2; see electronic supplementary material, figure S3). At higher elevation WSR stations located within the Appalachian Mountains, migratory birds were detected at all three altitudes. At lower elevation WSR stations, migratory birds were detected primarily at the lowest migration altitude. The regression tree analyses identified relationships between the density of migrants and wind velocity at low (*R*^2^=0.44) and intermediate (*R*^2^=0.58) migration altitudes (figure 4). There was no evidence for differences among years or that wind bearing was an important predictor of migration density. No variables were identified as important in predicting the density of migrants at high migration altitudes. At low migration altitudes, higher migration densities were associated with lower wind velocities at the end of the season (after 2 November; figure 4*a*). During the beginning of the season (before 15 September), the densities of migrants at lower migration altitudes presented no relationship with wind velocity (figure 4*a*). During the middle of the season, higher migration densities at low migration altitudes were associated with lower wind velocities (figure 4*a*). At intermediate migration altitudes, migration density was related to wind velocity during the middle of the season (between 6 November and 7 September); here, higher migration densities were associated with lower wind velocities (figure 4*b*).

**Figure 5.**
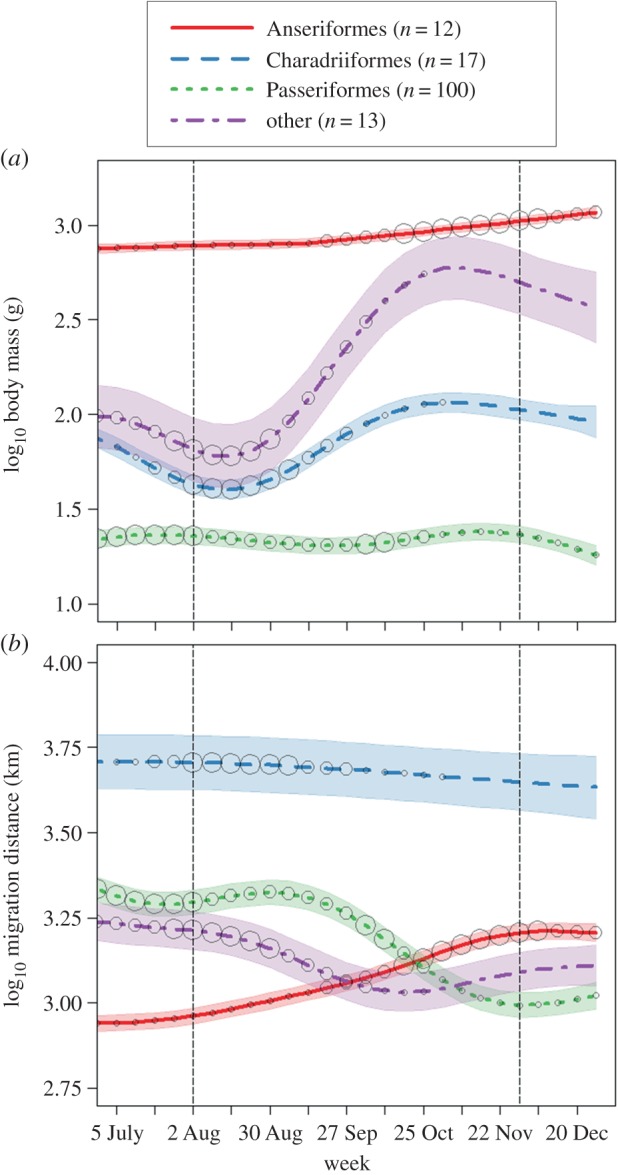
Weekly assemblage-level estimates of (*a*) body size and (*b*) total migration distance at 12 weather surveillance radar (WSR) stations located in the northeastern USA (see the electronic supplementary material, figure S1) for 142 species of nocturnal migrating birds classified by order. The ‘other’ category contains 13 species in eight additional orders. The vertical dashed lines indicate the beginning and end of the radar data period. Regression lines and shaded areas show the fitted response and 95% confidence bands of generalized additive mixed models with WSR station as a random effect. The size of the circle displays the weekly average probability of occurrence for species at the WSR stations, scaled within each avian order from low (small circle) to high (large circle) probability of occurrence.

The weekly estimates of diurnal probability of occurrence of the 142 nocturnally migrating species (see the electronic supplementary material, table S1) described changes in species composition as the migration season progressed (figure 5). In general, smaller bodied species were detected at higher probabilities at the WSR stations early in the season, and larger bodied species were detected at higher probabilities at the WSR stations late in the season (figure 5*a*). In the majority of cases, species with longer migration distances were detected at higher probabilities early in the season, and species with shorter migration distances were detected at higher probabilities late in the season (figure 5*b*).

**Figure 6.**
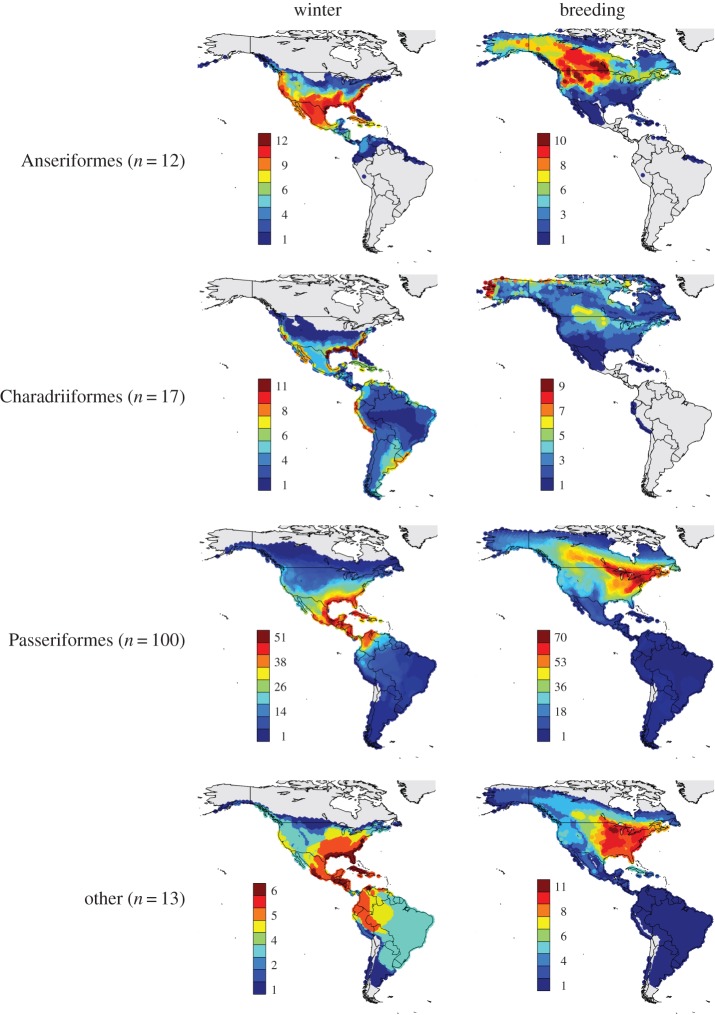
Maps of species richness within the Western Hemisphere based on winter and breeding distributions for 142 species of nocturnal migrating birds classified by order. The ‘other’ category contains 13 species in eight additional orders. Species richness was estimated by combining seasonal range maps [32] whose polygons were converted to cells of an equal-area icosahedron having a spatial resolution of 12 452 km^2^.

When examined by avian order, only species of Anseriformes, representing the largest-bodied migrants in the region, were detected at higher probabilities late in the season (figure 5*a*). Early in the season, Charadriiformes, the longest distance migrants in the region (figure 6), had higher probabilities of occurrence (figure 5*b*). Passeriformes that were long-distance migrants also had higher probabilities of occurrence early in the season (figure 5*b*). Passeriformes that had intermediate migration distances had higher probabilities of occurrence during the middle of the season (figure 5*b*). Thus, only Anseriformes, the largest-bodied migratory species in the region having intermediate migration distances (figure 6) had the highest probabilities of occurrence at the end of the season (figure 5).

## 4. Discussion

Altitudinal distributions of nocturnally migrating birds during autumn migration at mid-latitudes in North America presented seasonal patterns with distinct temporal and spatial structure. Contrary to expectations, the presence of progressively stronger westerly winds at higher migration altitudes did not result in systematic declines in migration altitudes. Rather, the median migration altitude remained relatively consistent across the season, and the overall distribution of migration altitudes was in agreement with earlier radar-based assessments conducted in the region [23]. In addition, our findings provide strong evidence for differences in the night-to-night variability in the altitudes at which migrants are flying. Migration altitudes were narrowly defined during the middle of the season, and broadly defined at the beginning and especially at the end of the season. This heteroscedasticity resulted in distributions of migration altitudes whose lower boundary was weakly convex and whose upper boundary was strongly concave in form. This seasonal variation was accompanied by different associations with wind velocity but not wind bearing. The density of migrants was generally associated with weaker winds, as previously identified for this system [16]. However, these associations were most evident at low to intermediate migration altitudes during the end and especially the middle of the season. This seasonal variation in the altitudinal distribution was also accompanied by changes in the composition of migratory species. The broader altitudinal associations documented early in the season were related to the passage of small- to intermediate-sized long-distance migrants in the orders Charadriiformes and Passeriformes, and late in the season by the passage of large-bodied migrants in the order Anseriformes having intermediate migration distances. Thus, seasonality in the composition of migratory species, and related variation in migration strategies and behaviour, may have given rise to the convex–concave bounded distribution of migration altitudes.

The small- to intermediate-sized long-distance migrants that occurred early in the season are species that, owing to their smaller body sizes, can climb to higher migration altitudes more efficiently [17,18]. The lower air resistance at these altitudes may facilitate more efficient, long-distance migratory flights for these species [19] and the presence of milder atmospheric conditions at mid-latitudes early in the season may further enhance these movements, which may explain the lack of an early-season association with milder atmospheric conditions. Another explanation may originate from the stronger time selection pressures that long-distance migrants operate under, which can result in associations with less favourable or a broader range of atmospheric conditions during migration [36]. Our findings indicate that these early-season migrants include Charadriiformes (shorebirds), which are intermediate in body size and have the longest migration distances, and Passeriformes (passerines), which are small bodied and have more variable migration distances (figure 6; electronic supplementary material, table S1). Many Charadriiformes and several Passeriformes travel from Arctic breeding grounds to the Atlantic coast of North America in the autumn, from which they undertake transoceanic flights to wintering grounds in the Caribbean or South America [23,37,38].

Our findings suggest that the broad altitudinal associations observed late in the migration season, primarily at inland sites, are due to the migration of Anseriformes (waterfowl). Anseriformes are the largest nocturnal migrants within the region with breeding and wintering grounds located primarily within North America, with a large number of species wintering along the Atlantic coastline (figure 6). These seasonal geographical patterns provide an explanation for the greater prevalence of late-season high-altitude migrants within the interior of the study area and not along the Atlantic coastline. Thus, our findings suggest large-bodied Anseriformes are expending additional energy to climb to higher migration altitudes [17,18], even though their migration journeys are relatively short and atmospheric conditions late in the season at higher migration altitudes are more likely to contain strong westerly winds (figure 1). However, westerly winds in this case may provide tailwind support for species travelling to wintering grounds along the Atlantic coastline. In total, our findings suggest Anseriformes affect the breadth of late-season migration altitudes within the region. These patterns and associated drivers are probably worth exploring in more detail.

Under global climate change [39], temperatures in the Arctic are warming more rapidly than at the equator [40,41]. This phenomenon, termed Arctic amplification, has the potential to alter atmospheric circulation patterns including the seasonal behaviour of the polar-front jet stream. Specifically, the development of a weaker temperature gradient between the Arctic and mid-latitudes is expected to weaken the jet stream and increase meridional flow. Although a causal relationship has been difficult to demonstrate, current evidence suggests Arctic amplification has increased the frequency, intensity and persistence of climate extremes at mid-latitudes, especially during the autumn and winter [42–46]. For birds migrating through the mid-latitudes in the autumn, these changes may result in stronger and more persistent westerly winds [43,46], which has the potential to interfere with some current migration strategies [47]. Based on our findings, we expect the frequency and quality of suitable atmospheric conditions for migratory flight to decrease under climate change, especially late in the season. This may be more pronounced within higher elevation regions where migrants are constrained to higher altitudes.

Possible approaches migrants could employ to counter these climate change related effects include changes in migration timing or changes in the choice of migration altitudes or routes. However, these approaches may only be relevant for short-distance migrants whose migration strategies tend to be more flexible and adaptive [48–50]. The more rigid migration strategies displayed by long-distance migrants, by contrast, may limit the application of these approaches, which could increase the chances of mortality during migration [51–53]. These approaches may also contain trade-offs that may reduce their overall effectiveness. For example, migrants may be able to avoid more extreme atmospheric conditions by shifting to earlier departure times, but this strategy has the potential to interfere with breeding and stopover activities. Similarly, the use of lower migration altitudes may allow migrants to avoid the presence of more extreme atmospheric conditions, but this approach has the potential to increase the overall cost of migration, especially for long-distance migrants. This approach may provide little benefit for species that migrate over mountains, and may increase the frequency of collisions with anthropogenic structures such as buildings [54] or communication towers [55]. Finally, changes in the location of migration routes may result in longer migration journeys and reduced access to higher quality stopover habitats.

The combination of data sources used in this study offers a unique empirical basis to assess how migration patterns and associations are defined across altitudes within a geographical region. However, for the majority of migratory species considered in this study, the northeastern USA encompasses a small component of their overall migration journey. Research that advances the methods used in this study to explore how patterns and associations are defined within complete migration flyways [56] across the full annual cycle [57] would be valuable. In addition, the ability of our eBird-based methods to accurately approximate seasonal trends in the composition of migratory species requires broader consideration and validation. Lastly, examining how seasonal changes in atmospheric conditions [56] and ecological productivity [27] act in concert to determine the timing and location of migration would be beneficial. By refining and expanding our empirical perspective, we will advance our understanding on how broad-scale migration strategies are structured and maintained, which will improve the realism of current projections of the implications of climate change for migratory bird populations [58]. In total, these efforts will promote more robust and comprehensive research perspectives and conservation policies where all aspects of the complex life cycles of these species will be considered.

## Supplementary Material

Table S1. The 142 species of nocturnal migratory birds considered in the analysis, their total-migration distance, and body mass.

## Supplementary Material

Figure S1. The geographic location and elevation of the 12 weather surveillance radar stations. Figure S2. Altitudes above sea level at which atmospheric conditions were estimated at the 12 weather surveillance radar stations. Figure S3. The fit of weighted generalized additive mixed models of the altitude of nocturnal migratory birds above sea level and above ground level at the 12 weather surveillance radar stations.

## Data accessibility

Data are available from the Dryad Digital Repository: http://dx.doi.org/10.5061/dryad.nq648.
