# Supplementary material for: Seasonal changes in the altitudinal distribution of nocturnally migrating birds during autumn migration
Source: R Soc Open Sci. 2015 Dec 9;2(12):150347. doi: 10.1098/rsos.150347 (PMC4807445; doi:10.1098/rsos.150347)
Supplement: Table S1. The 142 species of nocturnal migratory birds considered in the analysis, their total-migration distance, and body mass. [file rsos150347supp1.docx]

**Electronic supplementary material**

**Table S1.** The 142 species of nocturnal migratory birds considered in the analysis, their total-migration distance, and body mass.

| Common name | Scientific name | Order | Family | Distance (km) | Mass (g) |
| --- | --- | --- | --- | --- | --- |
| Ross's Goose | *Chen rossii* | Anseriformes | Anatidae | 3253 | 1632 |
| Wood Duck | *Aix sponsa* | Anseriformes | Anatidae | 796 | 681 |
| American Wigeon | *Anas americana* | Anseriformes | Anatidae | 3084 | 792 |
| American Black Duck | *Anas rubripes* | Anseriformes | Anatidae | 1492 | 1400 |
| Blue-winged Teal | *Anas discors* | Anseriformes | Anatidae | 3564 | 380 |
| Northern Shoveler | *Anas clypeata* | Anseriformes | Anatidae | 3317 | 636 |
| Northern Pintail | *Anas acuta* | Anseriformes | Anatidae | 2821 | 1006 |
| Green-winged Teal | *Anas crecca* | Anseriformes | Anatidae | 2188 | 359 |
| Canvasback | *Aythya valisineria* | Anseriformes | Anatidae | 2830 | 1252 |
| Ring-necked Duck | *Aythya collaris* | Anseriformes | Anatidae | 2094 | 730 |
| Lesser Scaup | *Aythya affinis* | Anseriformes | Anatidae | 3299 | 850 |
| Ruddy Duck | *Oxyura jamaicensis* | Anseriformes | Anatidae | 1321 | 629 |
| Great Blue Heron | *Ardea herodias* | Pelecaniformes | Ardeidae | 854 | 2748 |
| Great Egret | *Ardea alba* | Pelecaniformes | Ardeidae | 192 | 935 |
| Black-bellied Plover | *Pluvialis squatarola* | Charadriiformes | Charadriidae | 6335 | 250 |
| American Golden-Plover | *Pluvialis dominica* | Charadriiformes | Charadriidae | 12047 | 149 |
| Killdeer | *Charadrius vociferus* | Charadriiformes | Charadriidae | 1406 | 92 |
| Greater Yellowlegs | *Tringa melanoleuca* | Charadriiformes | Scolopacidae | 7705 | 162 |
| Willet | *Tringa semipalmata* | Charadriiformes | Scolopacidae | 3368 | 233 |
| Upland Sandpiper | *Bartramia longicauda* | Charadriiformes | Scolopacidae | 9286 | 154 |
| Hudsonian Godwit | *Limosa haemastica* | Charadriiformes | Scolopacidae | 12953 | 222 |
| Marbled Godwit | *Limosa fedoa* | Charadriiformes | Scolopacidae | 2478 | 326 |
| Red Knot | *Calidris canutus* | Charadriiformes | Scolopacidae | 8711 | 142 |
| Semipalmated Sandpiper | *Calidris pusilla* | Charadriiformes | Scolopacidae | 7794 | 28 |
| Western Sandpiper | *Calidris mauri* | Charadriiformes | Scolopacidae | 6062 | 27 |
| Least Sandpiper | *Calidris minutilla* | Charadriiformes | Scolopacidae | 6370 | 22 |
| Baird's Sandpiper | *Calidris bairdii* | Charadriiformes | Scolopacidae | 11752 | 39 |
| Dunlin | *Calidris alpina* | Charadriiformes | Scolopacidae | 4113 | 50 |
| Long-billed Dowitcher | *Limnodromus scolopaceus* | Charadriiformes | Scolopacidae | 5593 | 100 |
| American Woodcock | *Scolopax minor* | Charadriiformes | Scolopacidae | 951 | 176 |
| Wilson's Phalarope | *Phalaropus tricolor* | Charadriiformes | Scolopacidae | 9418 | 52 |
| Yellow-billed Cuckoo | *Coccyzus americanus* | Cuculiformes | Cuculidae | 6061 | 64 |
| Black-billed Cuckoo | *Coccyzus erythropthalmus* | Cuculiformes | Cuculidae | 5696 | 51 |
| Common Nighthawk | *Chordeiles minor* | Caprimulgiformes | Caprimulgidae | 7099 | 79 |
| Chuck-will s-widow | *Antrostomus carolinensis* | Caprimulgiformes | Caprimulgidae | 1353 | 109 |
| Eastern Whip-poor-will | *Antrostomus vociferus* | Caprimulgiformes | Caprimulgidae | 1940 | 50 |
| Chimney Swift | *Chaetura pelagica* | Apodiformes | Apodidae | 5028 | 24 |
| Ruby-throated Hummingbird | *Archilochus colubris* | Apodiformes | Trochilidae | 2757 | 3 |
| Belted Kingfisher | *Megaceryle alcyon* | Coraciiformes | Alcedinidae | 1601 | 148 |
| Red-headed Woodpecker | *Melanerpes erythrocephalus* | Piciformes | Picidae | 424 | 72 |
| Yellow-bellied Sapsucker | *Sphyrapicus varius* | Piciformes | Picidae | 2761 | 50 |
| Northern Flicker | *Colaptes auratus* | Piciformes | Picidae | 865 | 135 |
| Olive-sided Flycatcher | *Contopus cooperi* | Passeriformes | Tyrannidae | 7108 | 32 |
| Eastern Wood-Pewee | *Contopus virens* | Passeriformes | Tyrannidae | 4906 | 14 |
| Yellow-bellied Flycatcher | *Empidonax flaviventris* | Passeriformes | Tyrannidae | 4018 | 12 |
| Acadian Flycatcher | *Empidonax virescens* | Passeriformes | Tyrannidae | 3467 | 13 |
| Alder Flycatcher | *Empidonax alnorum* | Passeriformes | Tyrannidae | 7299 | 13 |
| Willow Flycatcher | *Empidonax traillii* | Passeriformes | Tyrannidae | 4457 | 13 |
| Least Flycatcher | *Empidonax minimus* | Passeriformes | Tyrannidae | 3759 | 10 |
| Eastern Phoebe | *Sayornis phoebe* | Passeriformes | Tyrannidae | 1775 | 20 |
| Great Crested Flycatcher | *Myiarchus crinitus* | Passeriformes | Tyrannidae | 3070 | 32 |
| Eastern Kingbird | *Tyrannus tyrannus* | Passeriformes | Tyrannidae | 6602 | 40 |
| White-eyed Vireo | *Vireo griseus* | Passeriformes | Vireonidae | 1043 | 11 |
| Yellow-throated Vireo | *Vireo flavifrons* | Passeriformes | Vireonidae | 2840 | 18 |
| Blue-headed Vireo | *Vireo solitarius* | Passeriformes | Vireonidae | 2736 | 15 |
| Warbling Vireo | *Vireo gilvus* | Passeriformes | Vireonidae | 2790 | 13 |
| Philadelphia Vireo | *Vireo philadelphicus* | Passeriformes | Vireonidae | 4136 | 12 |
| Red-eyed Vireo | *Vireo olivaceus* | Passeriformes | Vireonidae | 5048 | 16 |
| Red-breasted Nuthatch | *Sitta canadensis* | Passeriformes | Sittidae | 560 | 10 |
| Brown Creeper | *Certhia americana* | Passeriformes | Certhiidae | 326 | 8 |
| House Wren | *Troglodytes aedon* | Passeriformes | Troglodytidae | 1253 | 11 |
| Sedge Wren | *Cistothorus platensis* | Passeriformes | Troglodytidae | 835 | 10 |
| Marsh Wren | *Cistothorus palustris* | Passeriformes | Troglodytidae | 1405 | 11 |
| Golden-crowned Kinglet | *Regulus satrapa* | Passeriformes | Regulidae | 1060 | 6 |
| Ruby-crowned Kinglet | *Regulus calendula* | Passeriformes | Regulidae | 2246 | 6 |
| Veery | *Catharus fuscescens* | Passeriformes | Turdidae | 7864 | 32 |
| Gray-cheeked Thrush | *Catharus minimus* | Passeriformes | Turdidae | 7631 | 33 |
| Bicknell's Thrush | *Catharus bicknelli* | Passeriformes | Turdidae | 3185 | 28 |
| Swainson's Thrush | *Catharus ustulatus* | Passeriformes | Turdidae | 6523 | 30 |
| Hermit Thrush | *Catharus guttatus* | Passeriformes | Turdidae | 2182 | 30 |
| Wood Thrush | *Hylocichla mustelina* | Passeriformes | Turdidae | 2735 | 48 |
| American Robin | *Turdus migratorius* | Passeriformes | Turdidae | 1165 | 78 |
| Gray Catbird | *Dumetella carolinensis* | Passeriformes | Mimidae | 2199 | 35 |
| Northern Mockingbird | *Mimus polyglottos* | Passeriformes | Mimidae | 37 | 48 |
| Brown Thrasher | *Toxostoma rufum* | Passeriformes | Mimidae | 946 | 69 |
| Blue-winged Warbler | *Vermivora cyanoptera* | Passeriformes | Parulidae | 2478 | 9 |
| Golden-winged Warbler | *Vermivora chrysoptera* | Passeriformes | Parulidae | 3694 | 9 |
| Tennessee Warbler | *Oreothlypis peregrina* | Passeriformes | Parulidae | 4888 | 9 |
| Orange-crowned Warbler | *Oreothlypis celata* | Passeriformes | Parulidae | 3155 | 9 |
| Nashville Warbler | *Oreothlypis ruficapilla* | Passeriformes | Parulidae | 3092 | 8 |
| Northern Parula | *Setophaga americana* | Passeriformes | Parulidae | 2234 | 8 |
| Yellow Warbler | *Setophaga petechia* | Passeriformes | Parulidae | 5143 | 10 |
| Chestnut-sided Warbler | *Setophaga pensylvanica* | Passeriformes | Parulidae | 3817 | 10 |
| Magnolia Warbler | *Setophaga magnolia* | Passeriformes | Parulidae | 3891 | 8 |
| Cape May Warbler | *Setophaga tigrina* | Passeriformes | Parulidae | 4463 | 10 |
| Black-throated Blue Warbler | *Setophaga caerulescens* | Passeriformes | Parulidae | 3056 | 10 |
| Yellow-rumped Warbler | *Setophaga coronata* | Passeriformes | Parulidae | 2627 | 12 |
| Black-throated Green Warbler | *Setophaga virens* | Passeriformes | Parulidae | 3671 | 9 |
| Blackburnian Warbler | *Setophaga fusca* | Passeriformes | Parulidae | 4885 | 10 |
| Yellow-throated Warbler | *Setophaga dominica* | Passeriformes | Parulidae | 1539 | 10 |
| Pine Warbler | *Setophaga pinus* | Passeriformes | Parulidae | 709 | 12 |
| Prairie Warbler | *Setophaga discolor* | Passeriformes | Parulidae | 1651 | 8 |
| Palm Warbler | *Setophaga palmarum* | Passeriformes | Parulidae | 3270 | 10 |
| Bay-breasted Warbler | *Setophaga castanea* | Passeriformes | Parulidae | 4867 | 12 |
| Blackpoll Warbler | *Setophaga striata* | Passeriformes | Parulidae | 7137 | 12 |
| Cerulean Warbler | *Setophaga cerulea* | Passeriformes | Parulidae | 4431 | 9 |
| Black-and-white Warbler | *Mniotilta varia* | Passeriformes | Parulidae | 3323 | 11 |
| American Redstart | *Setophaga ruticilla* | Passeriformes | Parulidae | 4571 | 8 |
| Prothonotary Warbler | *Protonotaria citrea* | Passeriformes | Parulidae | 2615 | 14 |
| Worm-eating Warbler | *Helmitheros vermivorum* | Passeriformes | Parulidae | 2110 | 13 |
| Ovenbird | *Seiurus aurocapilla* | Passeriformes | Parulidae | 3138 | 19 |
| Northern Waterthrush | *Parkesia noveboracensis* | Passeriformes | Parulidae | 5148 | 16 |
| Louisiana Waterthrush | *Parkesia motacilla* | Passeriformes | Parulidae | 2559 | 20 |
| Kentucky Warbler | *Geothlypis formosa* | Passeriformes | Parulidae | 2387 | 14 |
| Connecticut Warbler | *Oporornis agilis* | Passeriformes | Parulidae | 6560 | 13 |
| Mourning Warbler | *Geothlypis philadelphia* | Passeriformes | Parulidae | 4858 | 12 |
| Common Yellowthroat | *Geothlypis trichas* | Passeriformes | Parulidae | 2298 | 10 |
| Hooded Warbler | *Setophaga citrina* | Passeriformes | Parulidae | 2040 | 11 |
| Wilson's Warbler | *Cardellina pusilla* | Passeriformes | Parulidae | 4224 | 7 |
| Canada Warbler | *Cardellina canadensis* | Passeriformes | Parulidae | 5297 | 10 |
| Yellow-breasted Chat | *Icteria virens* | Passeriformes | Parulidae | 2576 | 24 |
| Eastern Towhee | *Pipilo erythrophthalmus* | Passeriformes | Emberizidae | 573 | 41 |
| American Tree Sparrow | *Spizella arborea* | Passeriformes | Emberizidae | 2456 | 19 |
| Chipping Sparrow | *Spizella passerina* | Passeriformes | Emberizidae | 1978 | 12 |
| Field Sparrow | *Spizella pusilla* | Passeriformes | Emberizidae | 469 | 12 |
| Vesper Sparrow | *Pooecetes gramineus* | Passeriformes | Emberizidae | 1789 | 26 |
| Lark Sparrow | *Chondestes grammacus* | Passeriformes | Emberizidae | 1230 | 29 |
| Savannah Sparrow | *Passerculus sandwichensis* | Passeriformes | Emberizidae | 2595 | 21 |
| Grasshopper Sparrow | *Ammodramus savannarum* | Passeriformes | Emberizidae | 1498 | 17 |
| Henslow's Sparrow | *Ammodramus henslowii* | Passeriformes | Emberizidae | 996 | 13 |
| Seaside Sparrow | *Ammodramus maritimus* | Passeriformes | Emberizidae | 848 | 23 |
| Fox Sparrow | *Passerella iliaca* | Passeriformes | Emberizidae | 2803 | 33 |
| Song Sparrow | *Melospiza melodia* | Passeriformes | Emberizidae | 939 | 23 |
| Lincoln's Sparrow | *Melospiza lincolnii* | Passeriformes | Emberizidae | 2725 | 17 |
| Swamp Sparrow | *Melospiza georgiana* | Passeriformes | Emberizidae | 1961 | 16 |
| White-throated Sparrow | *Zonotrichia albicollis* | Passeriformes | Emberizidae | 1933 | 24 |
| White-crowned Sparrow | *Zonotrichia leucophrys* | Passeriformes | Emberizidae | 2552 | 28 |
| Dark-eyed Junco | *Junco hyemalis* | Passeriformes | Emberizidae | 1454 | 20 |
| Rose-breasted Grosbeak | *Pheucticus ludovicianus* | Passeriformes | Cardinalidae | 4061 | 42 |
| Blue Grosbeak | *Passerina caerulea* | Passeriformes | Cardinalidae | 1712 | 28 |
| Indigo Bunting | *Passerina cyanea* | Passeriformes | Cardinalidae | 2351 | 15 |
| Dickcissel | *Spiza americana* | Passeriformes | Cardinalidae | 4023 | 28 |
| Bobolink | *Dolichonyx oryzivorus* | Passeriformes | Icteridae | 8108 | 34 |
| Eastern Meadowlark | *Sturnella magna* | Passeriformes | Icteridae | 389 | 104 |
| Orchard Oriole | *Icterus spurius* | Passeriformes | Icteridae | 2638 | 19 |
| Baltimore Oriole | *Icterus galbula* | Passeriformes | Icteridae | 3340 | 34 |
| Summer Tanager | *Piranga rubra* | Passeriformes | Cardinalidae | 3452 | 29 |
| Scarlet Tanager | *Piranga olivacea* | Passeriformes | Cardinalidae | 5058 | 28 |
| Blue-gray Gnatcatcher | *Polioptila caerulea* | Passeriformes | Polioptilidae | 987 | 6 |
| Le Conte's Sparrow | *Ammodramus leconteii* | Passeriformes | Emberizidae | 2263 | 13 |
| Clay-colored Sparrow | *Spizella pallida* | Passeriformes | Emberizidae | 3025 | 11 |
| Winter Wren | *Troglodytes hiemalis* | Passeriformes | Troglodytidae | 1125 | 10 |
