# Supplementary material for: Seasonal changes in the altitudinal distribution of nocturnally migrating birds during autumn migration
Source: R Soc Open Sci. 2015 Dec 9;2(12):150347. doi: 10.1098/rsos.150347 (PMC4807445; doi:10.1098/rsos.150347)
Supplement: Figure S1. The geographic location and elevation of the 12 weather surveillance radar stations. Figure S2. Altitudes above sea level at which atmospheric conditions were estimated at the 12 weather surveillance radar stations. Figure S3. The fit of weighted generalized additive mixed models of the a [file rsos150347supp2.docx]

**Electronic supplementary material**

**Figure S1.** The geographic location of the 12 weather surveillance radar (WSR) stations in the northeastern USA whose reflectivity data was considered in the analysis. The 12 WSR stations are located at the following elevations: 490 m (KBGM), 36 m (KBOX), 211 m (KBUF), 733 m (KCCX), 97 m (KCXX), 45 m (KDIX), 15 m (KDOX), 557 m (KENX), 125 m (KGYX), 83 m (KLWX), 26 m (KOKX), and 563 m (KTYX) above sea level.

**Figure S2.** Altitudes above sea level (ASL) at which atmospheric conditions were estimated, averaged across the locations of 12 weather surveillance radar (WSR) stations in the northeastern USA (see the electronic supplementary material, figure 1). Atmospheric conditions were measured at 3-hourly intervals at three isobaric levels during the period August 1^st^ to November 30^th^ for the years 2010 (green) and 2011 (blue).

**Figure S3.** The fit of weighted generalized additive mixed models of the altitude of nocturnal migratory birds above sea level (ASL; top row) and above ground level (AGL; bottom row) at 12 weather surveillance radar (WSR) stations located in the northeastern USA (see the electronic supplementary material, figure 1) estimated daily during the autumn of 2010 and 2011. The fits are applied to three quantile levels organized by column (*τ* = 0.01, 0.50, 0.99). Each fit contains a point-wise bootstrap 90% confidence interval. Reflectivity is the weighting factor in each model, and year is included as a random effect in each model. The dotted lines in the ASL plots (top row) are the three altitudes considered in the regression tree analysis (762, 1700, and 2734 m ASL). The shade of the fitted lines in each plot indicates low (light) to high (dark) elevations of the WSR stations.
